# Brain’s functional network clustering coefficient changes in response to instruction (RTI) in students with and without reading disabilities: Multi-leveled reading brain’s RTI

**DOI:** 10.1080/23311908.2018.1424680

**Published:** 2018-01-09

**Authors:** Todd L. Richards, Virginia W. Berninger, Kevin Yagle, Robert D. Abbott, Dan Peterson

**Affiliations:** 1 Department of Radiology, Integrated Brain Imaging Center, University of Washington, Seattle, WA, USA; 2 Learning Sciences and Human Development, University of Washington, Seattle, WA, USA; 3 Educational Statistics and Measurement, University of Washington, Seattle, WA, USA

**Keywords:** reading disabilities, behavioral response to reading intervention, brain response to reading intervention, graph theory, correlations between white matter and gray matter clustering coefficients, Neuroscience, Language Teaching &, Learning, Radiology

## Abstract

In students in grades 4 to 9 (22 males, 20 females), two reading disability groups—dyslexia (*n* = 20) or oral and written language learning disability (OWL LD) (*n* = 6)—were compared to each other and two kinds of control groups—typical readers (*n* = 6) or dysgraphia (*n* = 10) on word reading/spelling skills and fMRI imaging before and after completing 18 computerized reading lessons. Mixed ANOVAs showed significant time effects on repeated measures within participants and between groups effects on three behavioral markers of reading disabilities—word reading/spelling: All groups improved on the three behavioral measures, but those without disabilities remained higher than those with reading disabilities. On fMRI reading tasks, analyzed for graph theory derived clustering coefficients within a neural network involved in cognitive control functions, on a *word level* task the time × group interaction was significant in right medial cingulate; on a *syntax level* task the time × group interaction was significant in left superior frontal and left inferior frontal gyri; and on a *multi-sentence text level* task the time × group interaction was significant in right middle frontal gyrus. Three white matter-gray matter correlations became significant only after reading instruction: axial diffusivity in left superior frontal region with right inferior frontal gyrus during *word reading judgments*; mean diffusivity in left superior corona radiata with left middle frontal gyrus during *sentence reading judgments*; and mean diffusivity in left anterior corona radiata with right middle frontal gyrus during *multi-sentence reading judgments*. Significance of results for behavioral and brain response to reading instruction (RTI) is discussed.

## Graphical abstract

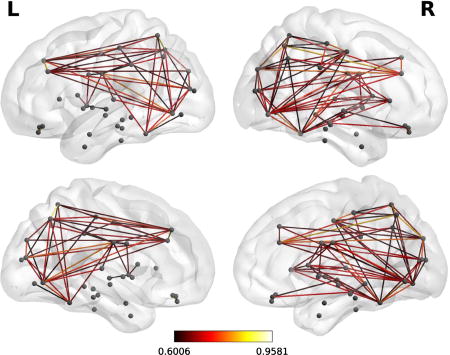

## 1. Introduction

Epidemiological studies have shown that among school-age children and youth not all reading disabilities are the same. Some reading disabilities are related to early emerging problems in oral language (Stoeckel et al., 2013). Some reading disabilities emerge at time of transition to school and formal reading instruction (Katusic, Colligan, Barbaresi, Schaid, & Jacobsen, 2001). Other specific learning disabilities involving written language acquisition (SLDs-WL) do not even involve reading (Katusic, Colligan, Weaver, & Barbaresi, 2009). Developmental research has shown that early emerging oral language disabilities during the preschool years may be related to late talking and/or late combining of words into syntactic structures that persist during the school years along with reading disabilities (Catts & Kamhi, 2005; Paul, Murray, Clancy, & Andrews, 1997; Scott, 2004; Thal, Bates, Goodman, & Jahn-Samilo, 1997). Accordingly, these specific learning disabilities have been referred to as oral and written language learning disabilities (OWL LD) or specific language impairment (SLI) in the research literature. However, SLI, for which syntactic skills are a hallmark impairment, is not the same as dyslexia, for which word level skills are a hallmark impairment (Catts, Adlof, Hogan, & Weismer, 2005). However, dyslexia also involves related subword skills such as phonological and orthographic awareness related to word decoding of pseudowords, oral and silent reading of real words, and spelling (Cao, Bitan, Chou, Burman, & Booth, 2006; Lefly & Pennington, 1991). Importantly, not all SLDs-WL even involve reading; some impair writing but not reading (Berninger, Nielsen, Abbott, Wijsman, & Raskind, 2008).

Thus, in the current study two kinds of controls were included for comparison with the two kinds of reading disabilities related to OWL LD or dyslexia—namely dysgraphia (impaired handwriting which may affect spelling but not word reading) and typical oral and written language learners. Although much prior brain research on reading has focused on a single level of language, for example, lexical (single word, Booth et al., 2003), syntax (single sentence, Caplan, 2004), or text (multiple sentences, Aboud, Bailey, Petrill, & Cutting, 2016), the current study was designed to include fMRI connectivity measures for reading at the word, sentence, and text levels of language.

Prior research had shown that early in schooling reading disabilities are related to anomalies in neural network connectivity (Hosseini et al., 2013). Of interest was whether that would also be the case later in schooling in students with persisting reading disabilities despite earlier intervention. Prior research had compared functional connectivity in children with dyslexia who varied in the nature of the prior specialized instruction they had received: no remediation, remediation of their reading problems only, or remediation of their reading and spelling problems. In contrast, the current study focused on changes, from before to after receiving specialized instruction. This instruction was aimed at not only word-level oral reading and word-specific spelling relevant to silent reading but also at the other levels of language that draw on word level skills—namely sentence syntax and multi-sentence text. Unlike prior research that compared word level and text level language processes in reading comprehension in adolescents who varied along a continuum in reading ability (Aboud et al., 2016), the current research compared groups of students during middle childhood and early adolescence who had undergone differential diagnosis for specific kinds of reading disabilities.

### 1.1. Evidence-based assignment to diagnostic groups

Diagnostic assessment was based on a comprehensive review of research across research groups and populations of developing language learners (Silliman & Berninger, 2011). For recent studies validating the procedures employed for diagnostic assessment, see Berninger, Richards, and Abbott (2015) and Sanders, Abbott, and Berninger (2017). These procedures included meeting criteria for

- typical learner without a specific learning disability (no evidence on assessment measures or parent reported history of any oral or written language learning disabilities) or
- dysgraphia (impaired handwriting on at least two measures, a learning profile without reading disabilities, and history of past and persisting handwriting problems despite intervention) or
- dyslexia (impaired word reading or decoding and spelling on at least two measures, a learning profile without oral language disabilities at the syntax or text level, and history of past and persisting word reading/decoding or spelling problems despite intervention) or
- oral and written language learning disability (OWL LD) (impaired listening comprehension, oral expression, reading comprehension, and/or written expression on at least two measures; early emergence of oral language problems during the preschool years and persisting oral and written language problems during the school years despite intervention).

For both typically developing language learners (Niedo, Abbott, & Berninger, 2014) and those with dysgraphia, dyslexia, or OWL LD (Sanders et al., 2017), Verbal Comprehension Index (VCI) on a widely used normed measure of intellectual ability (Wechsler, 2003) explained significant variance in writing and reading achievement. Likewise, cross-site samples of reading disabilities provided converging evidence for common brain bases related to VCI, which is sometimes estimated from the vocabulary subtest contributing to it (Eckert et al.,2016). None of these findings support defining SLDs on the basis of discrepancy between Full Scale Scores on the Wechsler Scales and reading or writing achievement. However, the evidence-based procedures used in the current study based on Berninger et al. (2015) required that the VCI scores on the Wechsler Scale (Wechsler, 2003) fall at least within the lower limit of the normal range (−1 1/3 SD or standard score of 80) to rule out possible or probable intellectual disability.

In addition, there was a further requirement for inclusion into an SLD diagnostic group. There had to be evidence that the hallmark impairment for an SLD diagnostic group had been persisting during middle childhood and early adolescence despite early literacy intervention. Leonard, Eckert, Given, Berninger, and Eden (2006) studied students with persisting reading problems who were not treatment responders for early intervention in reading and identified brain differences between those who were not treatment responders and those in an earlier study who responded readily to early intervention. Furthermore, assignment to diagnostic group in the current sample was based on learning profiles for levels of language in hallmark reading impairment in dysgraphia (none), dyslexia (lexical involved in oral word reading for real or pseudowords and word-specific spelling for silent word reading), or OWL LD (syntax involved in reading comprehension) or typical readers (controls). Sanders et al. (2017) had shown that these reading profiles for these groups contributed uniquely to reading achievement over and beyond verbal comprehension and the multiple components of working memory supporting language learning.

### 1.2. Research design and goals

After assignment to diagnostic groups, the participants completed the first brain imaging session and then were enrolled in an instructional intervention study. A unique feature of the instruction was that the computerized lessons were aimed at all levels of language close in time to create a functional reading system (Tanimoto, Thompson, Berninger, Nagy, & Abbott, 2015). These levels included *subword* level grapheme-phoneme correspondences and prefixes and suffixes for transforming base words, *word* level decoding and spelling, *syntax* level comprehension, and *text-level* reading of source material. The learning activities at the word level emphasized interrelationships among phonology, orthography, and morphology in the reading direction for English, which is a morphophonemic orthography (Cahill, Tiberius, & Herring, 2013; Nunes & Bryant, 2009; Nunes, Bryant, & Bindman, 1997; Venezky, 1970, 1999). The learning activities at the syntax level emphasized content and function words and word order in understanding sentence syntax. The learning activities at the text level involved reading source material to take notes and write summaries. Thus, the lessons were designed to include not only learning activities for impaired reading skills for dyslexia and OWL LD but also to facilitate reading development of typically developing readers and those with dysgraphia who may also benefit from teaching to all levels of language to create a functional reading system. After completing the 18 computerized lessons, the participants completed a second imaging session and reassessment of their reading skills to measure their response to instruction (RTI).

The goals of the current study were, therefore, twofold: to investigate both behavioral RTI and brain RTI. Simply analyzing RTI at the behavioral level or comparing those with reading disability in general with those without reading disability may not fully document whether, and if so, how the brain responds to reading instruction. In contrast to much prior research on the brain’s response to reading instruction in students with and without dyslexia, which had analyzed changes in a region of interest (ROI) (e.g. Aylward et al., 2003) or profiles of regional brain activation (e.g. Simos et al., 2007), the current study applied complex network analysis based on graph theory analysis (He & Evans, 2010) to evaluate the reading brain’s RTI following specialized reading instruction. To apply complex network analysis/graph theory analysis, as described in Rubinov and Sporns (2010), graphs were constructed of specific functional networks of documented significant statistical magnitude and theoretical interest based on prior research as explained in the methods.

Graph theory-based approaches model the brain as a complex network represented by a collection of nodes connected by edges. In this connectivity virtual graph, nodes indicate anatomical elements (e.g. brain regions) and edges represent the relationships between nodes (e.g. connectivity). After the network modeling procedure, various graph theoretical metrics can be used to investigate the organizational structure underlying the relevant networks. The graph-based network analyses support (a) visualization of the overall connectivity pattern among all the elements of the brain (e.g. brain regions), (b) quantitative characterization of the global organization, (c) inspection of the topological reconfiguration of the brain in response to resting conditions (Wang, Wang, et al., 2009) or external task modulation (Bassett et al., 2009; Eguiluz, Chialvo, Cecchi, Baliki, & Apkarian, 2005; Micheloyannis et al., 2009; Pachou et al., 2008; Wang, Zuo, & He, 2010) or pathological attacks (for reviews, see Bassett & Bullmore, 2009 and He, Chen, Gong, & Evans, 2009); (d) provision of a vital framework to elucidate the relationship between brain structure and function (Honey, Thivierge, & Sporns, 2010) in brain networks that have been demonstrated to organize intrinsically as modular small-world architectures capable of efficiently transferring information at a low wiring cost as well as exhibiting highly connected hub regions (Chen, He, Rosa-Neto, Germann, & Evans, 2008; Chen et al., 2008; Gong et al., 2009; Hagmann et al., 2008; He, Chen, & Evans, 2007; He, Chen, et al., 2009; Salvador et al., 2005); and (e) examination of the potential mechanisms involved in normal development (Fair et al., 2007, 2008, 2009; Supekar, Musen, & Menon, 2009), aging (Achard & Bullmore, 2007; Gong et al., 2009; Meunier, Achard, Morcom, & Bullmore, 2009; Micheloyannis et al., 2009; Wang et al., 2010), and various brain disorders (Buckner et al., 2009; He, Chen, Evans, 2008; He, Wang, et al., 2009; Liu et al., 2008; Stam, Jones, Nolte, Breakspear, & Scheltens, 2007; Supekar et al., 2009; Wang, Zhu, et al., 2009). In the current study applications of this methodology have been extended further to study of two kinds of reading disabilities (dyslexia and OWL LD) and two kinds of controls (with and without dysgraphia) during middle childhood and early adolescence.

### 1.3. Sets of research questions

#### 1.3.1. Set 1 research questions

Is there behavioral evidence that both groups with reading disabilities (dyslexia and OWL LD/SLI) differ from both control groups (with and without dysgraphia) in word-level skills that are impaired in dyslexia and may or may not be in OWL LD/SLI? If so, did both the reading disabilities and control groups show behavioral improvement in word-level reading skills? Was there any evidence of significant interactions between time (change from before to after intervention) and diagnostic group on the word-level behavioral measures?

#### 1.3.2. Set 2 research questions

Is there brain evidence that both groups with reading disabilities (dyslexia and OWL LD/SLI) differ from both control groups (with and without dysgraphia) on an fMRI word-level reading task? On an fMRI sentence-syntax level reading task? On an fMRI multi-sentence text reading task? If so, did both the reading disabilities and control groups show changes in magnitude of connectivity in the fMRI word-level reading task, the fMRI sentence/syntax reading task, and the fMRI multi-sentence text reading task after instructional intervention? Was there any evidence of significant interactions between time (change from before to after intervention) and diagnostic group on any of the fMRI reading tasks?

#### 1.3.3. Set 3 research questions

Was there evidence of changes in correlations between white matter indicators of DTI diffusivity and gray matter clustering coefficients after the specialized reading instruction compared to before the intervention? That is, if there was evidence of gray matter brain response to intervention (RTI) was it related to white matter RTI? For these correlations DTI diffusivity measures were used that were shown in prior research to show significant time effects in a related study the participants had also completed (Richards, Berninger, Yagel, Abbott, & Peterson, 2017).

## 2. Methods

### 2.1. Ascertainment of participants

Children with persisting reading disabilities in grades 4 to 9 were recruited from local schools near the university where the research was conducted and the university institutional board (IRB) had approved the research with human participants. The research was conducted in compliance with IRB approved procedures and the ethical guidelines of the American Psychological Association. Following a phone screening, informed consent was obtained from parents and assent from their children for diagnostic assessment at the university for those who appeared to meet research criteria for inclusion.

### 2.2. Assignment to diagnostic groups

Because specific learning disabilities occur in otherwise typically developing individuals, exclusionary criteria included evidence of overall cognitive or language functioning outside the normal range or previously diagnosed developmental disabilities or severe neurological disorders. The specific criteria for each of the four diagnostic groups, based on the assessment at the university for which students were given normed measures and parents completed questionnaires about developmental, medical, and educational history, were as follows.

#### 2.2.1. Typical reader control

There was no evidence by parent report of past or current struggles with reading—oral or silent word reading or decoding or reading comprehension. There was no evidence on clinical normed measures of hallmark impairments in oral or silent word reading or decoding or reading comprehension.

#### 2.2.2. Dysgraphia

Parent reported past and current history of persisting struggles with handwriting but not with reading. On clinical normed measures there was evidence of hallmark impairments on at least two handwriting measures but not on reading measures.

#### 2.2.3. Dyslexia

Parent reported past and current history of persisting struggles with oral and silent word reading despite early intervention. On clinical normed measures there was evidence of hallmark impairments on at least two real word reading or pseudoword decoding or spelling measures (recognizing or producing correct spellings). Both on parent-reported past and current history and normed measures there could not be indicators of oral language disabilities at the syntax or text level either during the preschool or school years, for example, in listening comprehension or oral expression; however, the word identification problems may have interfered with reading comprehension. A co-occurring speech disorder was not an exclusion criterion if it did not interfere with understanding heard language or age appropriate use of vocabulary or syntax construction in oral expression.

#### 2.2.4. OWL LD

Parents reported past history of early emergence of oral language problems during the preschool years, and despite early intervention, persisting oral and written language problems during the school years involving syntax (and text) such as listening comprehension, oral expression, reading comprehension, and/or written expression. On clinical normed measures there was evidence of hallmark impairments on at least two oral or written language measures involving syntax or text. Both on parent reported past and current history and on normed measures there had to be evidence of hallmark impairments at the syntax level of language. However, consistent with the cascading levels of language differential diagnosis model (Berninger et al., 2015), co-occurring word reading and decoding problems characteristic of dyslexia were not exclusion criteria.

### 2.3. Sample characteristics

Altogether 42 upper elementary and middle school participants (22 males and 20 females; grades 4 to 9; average age 11 years 10 months) completed brain imaging before and after they completed 18 lessons of specialized writing instruction: controls (*n* = 6), dysgraphia (*n* = 10), dyslexia (*n* = 20), and OWL LD (*n* = 6). However, two of the OWL LD group were missing some fMRI connectivity or DTI data and could not be used in all analyses. Ethnicity was primarily European American (80.5%), but also Asian American (4.9%), and other/mixed (14.6%). Parents’ level of education ranged from less than high school (mothers, 0%; fathers 2.4%), to high school (2.4%, mothers; 2.4% fathers), to more than high school, less than college (7.1%, mothers; 9.5%, father), to college (42.9%, mothers, 33.3%, fathers), to more than college (4.7%, mothers; 9.5%, fathers).

### 2.4. Imaging

All scans were acquired at the Diagnostic Imaging Sciences Center in collaboration with the Integrated Brain Imaging Center and had Institutional Review Board approval. First diffusion tensor imaging (DTI) scans and then functional magnetic resonance imaging (fMRI) connectivity scans were obtained for all 42 children on a Philips 3 T Achieva scanner (release 3.2.2 with the 32-channel head coil) to obtain measures of white matter integrity and fMRI functional connectivity, respectively. Participants practiced lying still before entering the scanner and were instructed to lie still throughout the scanning. They also practiced the tasks before scanning and had to achieve 90% accuracy on them to continue participation to ensure that performance on the brain imaging tasks did not reflect inability to do a task.

Each participant was screened for MRI safety before entering the scanner. Physiological monitoring was performed using the Philips pulse oximeter placed on the left hand index finger for cardiac recording; and respiration was recorded using the Philips bellows system where the air-filled bellows pad was placed on the abdomen. Head-immobilization was aided using an inflatable head-stabilization system (Crania, Elekta).

#### 2.4.1. MRI data acquisition

The following MRI series were scanned: (1) 3-plane scout view with gradient echo pulse sequence: TR/TE 9.8/4.6 ms; Field of view 250 × 250 × 50 mm; acquisition time 30.3 s; (2) reference scan (used in parallel imaging) with gradient echo pulse sequence: TR/TE 4.0/0.75 ms; Field of View 530 × 530 × 300 mm; acquisition time 44.4 s; (3) Resting State fMRI scan with echo-planar gradient echo pulse sequence (single shot): TR/TE 2000/25 ms; Field of view 240 × 240 × 99 mm; slice orientation transverse, acquisition voxel size 3.0 × 3.08 × 3.0 mm; acquisition matrix 80 × 80 × 33; slice thickness 3.0, SENSE factor in the AP direction 2.3; epi factor 37; bandwidth in the EPI frequency direction 1933 Hz, SoftTone factor 3.5, sound pressure 6.1 dB, 180 dynamic scans; 5 dummy scans; fold over direction AP, acquisition time 6:14 min/s; (4) B0 field map imaging with gradient echo pulse sequence and 2 echos; TR/TE 11/6.3 ms; delta TE 1.0 ms; slice orientation transverse, Field of view 240 × 240 × 129 mm; voxel size 1.5 × 1.5 × 3.0 mm; acquisition matrix 160 × 160 × 43, output image magnitude and phase, acquisition time 2:29 min/s; (5) MPRAGE structural scan: TR/TE 7.7/3.5 ms, Field of view 256 × 256 × 176 mm, slice orientation sagittal, voxel size 1 × 1 × 1 mm, inversion pulse delay 1100 ms, Sense factor 2 in the AP direction, acquisition time 5:33 min/s; (6) diffusion tensor imaging with echo-planar spin-echo diffusion pulse sequence: TR/TE 8593/78 ms, slice orientation transverse, Field of view 220 × 220 × 128 mm, voxel size 2.2 × 2.2 × 2.0 mm, bvalues 0 and 1000, output images 1 bvalue at 0 and 32 bvalues at 1000 with 32 different diffusion vector non-colinear directions, SoftTone factor 4.0, sound pressure 3.1 dB, bandwidth in the EPI frequency direction 1557.7 Hz, epi factor 57, acquisition time 9:35.7 min/s; and (7) fMRI during the reading tasks: same parameters as with the Resting State fMRI described above except with dynamic scans 396, acquisition time 13:26 min/s.

#### 2.4.2. fMRI reading tasks

Participants were instructed to look at a fixation cross (no reading task) or to complete a specific reading task. To ensure continuous cognitive engagement, each reading task was presented with self-paced advancing of stimuli for two minutes; (total of 396 dynamic scans for all reading tasks).

#### 2.4.3. Word-level lexical judgments requiring knowledge of word-specific spellings in silent word reading (Ehri, 1980)

The participant is instructed to press yes if written word on screen is a correctly spelled real word, but press no if written word on screen is not a correctly spelled word, even though when pronounced it sounds like a real word. Example of yes item is “bus.” Example of no item, a homonym, is foil “eer.”

#### 2.4.4. Syntactic-level reading comprehension judgments of sentences with and without homonym foils

The participant is instructed to press yes if the sentence could be a real sentence that is meaningful because all the words are spelled correctly and make sense in the sentence, but press no if the sentence is not meaningful because all the words do not make sense in the sentence. Each sentence was presented for 3 s. The “no” items differed from the “yes” items by only one word which was a homonym foil. This is an example of a yes sentence: “The bee, which buzzes, can sting you.” This is an example of a no sentence: “The bee, witch buzzes, can sting you.”

#### 2.4.5. Multi-sentence reading comprehension text judgments

The participant is instructed to read each of the four sentences that will appear on the monitor one at a time and then press yes if the fifth sentence is true based on the four prior sentences read or no if it is false. Five written sentences are presented on the monitor one at a time (each presented for constant time interval). The last one is always a statement about the accumulating text so far that can be answered true (yes) or false (no).

Example set for a true response follows:

Sentence 1: John handed Bill a note.
Sentence 2: It was from Sarah.
Sentence 3: Sarah had written that she wanted to talk to Bill.
Sentence 4: Bill frowned when he read the note.
Sentence 5: True or False? (press key to answer) (True) Bill was not pleased with what Sarah had written.

Example set for a false response follows:

Sentence 1: Tomorrow is the day of the picnic.
Sentence 2: If it rains, the picnic will be cancelled.
Sentence 3: Amy listens to the weather report.
Sentence 4: She hopes it will rain.
Sentence 5: True or False? (press key to answer) (false) Amy wants to go to the picnic.

### 2.5. Data analyses

#### 2.5.1. Behavioral measures

Mixed ANOVA with repeated measures for time and between participant measures for diagnostic groups were used to evaluate targeted reading achievement measures related to the research questions underlying the current study rather than every measure in the assessment battery (for all group and individual participant findings for RTI, see Tanimoto et al., 2015). Results for the current study are reported for main effects for time and diagnostic groups and time × group interactions.

#### 2.5.2. Functional connectivity analyses

Functional images were corrected for motion using FSL MCFLIRT (Jenkinson, Bannister, Brady, & Smith, 2002), and then high-pass filtered at sigma = 20.83 (in units of fMRI volumes not seconds). Average Motion scores (as given in the MCFLIRT report) were computed for each participant and average motion score (mean absolute displacement) for each of the groups: control 1.31 ± 1.37 mm, dysgraphic 1.50 ± 1.23 mm, dyslexic 1.47 ± 1.03 mm, and OWL LD 1.32+/−0.638 mm. Spikes were identified and removed using the default parameters in AFNI’s 3dDespike. Slice-timing correction was applied with FSL’s slicetimer and spatial smoothing was performed using a 3D Gaussian kernel with FWHM = 4.0 mm. Time series motion parameters and the mean signal for eroded (1 mm in 3D) masks of the lateral ventricles and white matter (derived from running FreeSurfer’s recon-all on the T1-weighted image) were analyzed. Co-registration of functional images to the T1 image was performed using boundary-based registration based on a white matter segmentation of the T1 image through epi_reg in FSL. The MPRAGE structural scan was segmented using FreeSurfer software; white matter regressors were used to remove unwanted physiological components. Software was written in gfortran to compute a 68 × 68 correlation matrix which was used in the Brain Connectivity Toolbox. This 68 × 68 correlation matrix was made by finding the cross correlation between the fMRI time series signal between brain regions where each of the 68 brain regions were parceled using the cortical regions of the Mori atlas (Oishi, Faria, & Mori, 2010, May). The individual space of the fMRI scan was coregistered to this atlas using FSL FLIRT software.

To conduct Graph Theory Analysis, we used matlab software called “Brain Connectivity Toolbox,” https://sites.google.com/site/bctnet/construction to perform the complex network analysis/graph theory analysis as described in Rubinov and Sporns (2010). Clustering coefficients for the nodes in the Cingulate Operculum (CO) network (Horowitz-Kraus, Toro-Serey & DiFrancesco, 2015; Rubinov & Sporns, 2010) were calculated using this toolbox because Vaden et al. (2013) demonstrated the importance of the cingulo-opercular (CO) network in language processing. The CO network (a) supports cognitive control during task performance, and thought to detect errors in behavior and signal need for cognitive strategy adjustment (Dosenbach et al., 2007); (b) displays increased activity during the performance of many complex cognitive tasks (Dosenbach et al., 2006); (c) predicts cognitive performance (Rypma et al., 2006; Seeley et al., 2007; Song et al., 2008) involving top-down control during task performance (Dosenbach, Fair, Cohen, Schlaggar, & Petersen, 2008); (d) facilitates the maintenance of task-relevant goals; and (e) regulates behavioral adjustment based on error information (Cocchi, Zalesky, Fornito, & Mattingley, 2013).

Brain regions were identified based on the overlap of the 68 regions of interest in the Mori atlas with the cingulo-operculum network brain regions. Altogether eight regions of significant fMRI connectivity bilaterally were identified, all of which were previously shown to be significant after correction for multiple comparisons as processed using FSL software randomize: left and right cingulate gyrus, left and right superior frontal gyrus, left and right middle frontal gyrus, left and right inferior frontal gyrus, left and right superior temporal gyrus, left and right insula, left and right cingulum (cingulate gyrus), and left and right cingulum (hippocampus). Results reported are for clustering coefficient measures informed by graph theory, according to which a *clustering coefficient* is a measure of the degree to which nodes in a graph tend to cluster together. In most real-world networks, and in particular social networks, nodes tend to create tightly knit groups characterized by a relatively high density of mutual interconnections; this likelihood tends to be greater than the average probability of a tie randomly established between two nodes (Holland & Leinhardt, 1971).

**Figure 1:**
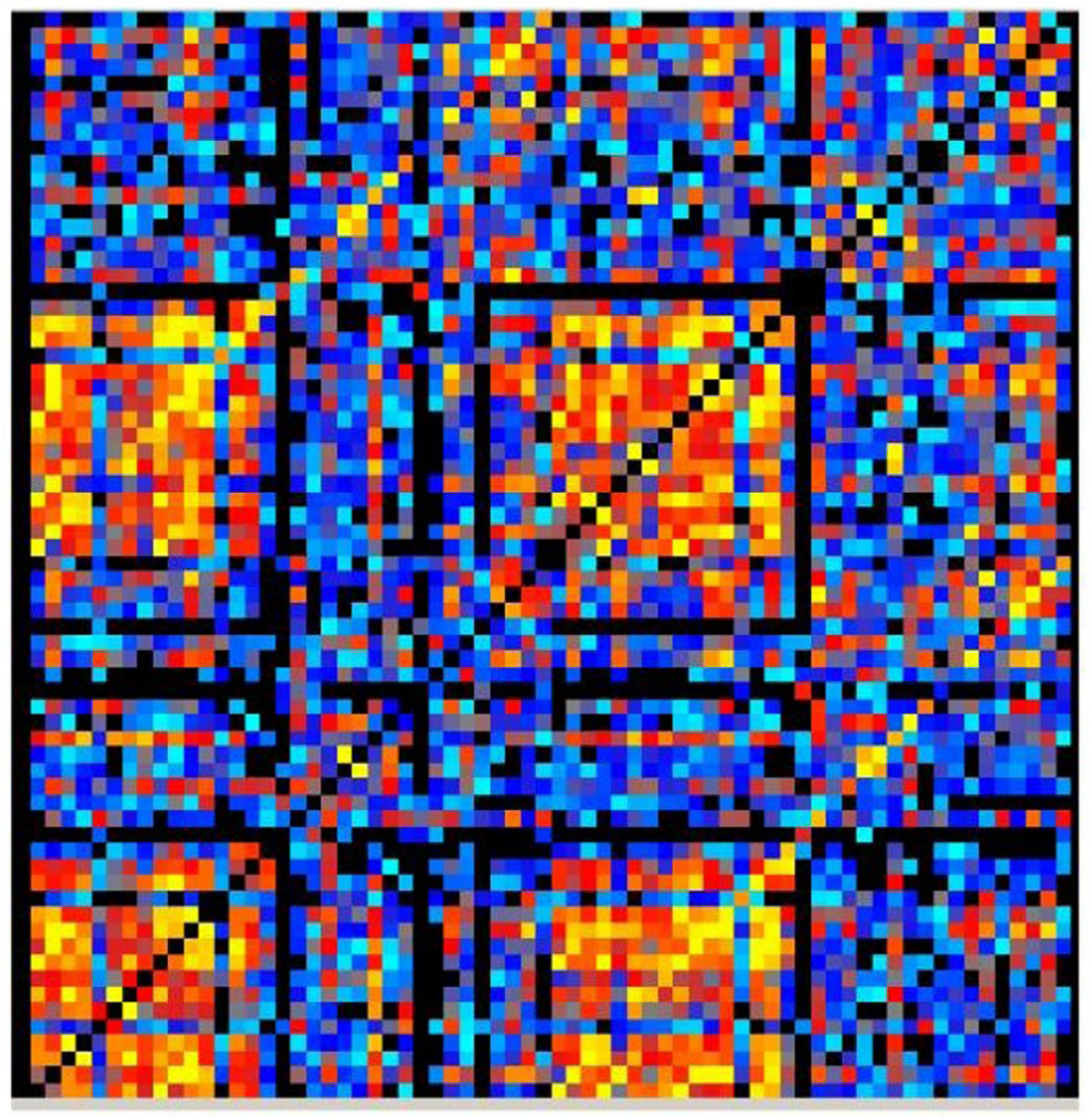
A correlation matrix map of 68 × 68 dimension made from the 68 cortical brain regions during the multiple sentence reading task of a control participant.

**Figure 2:**
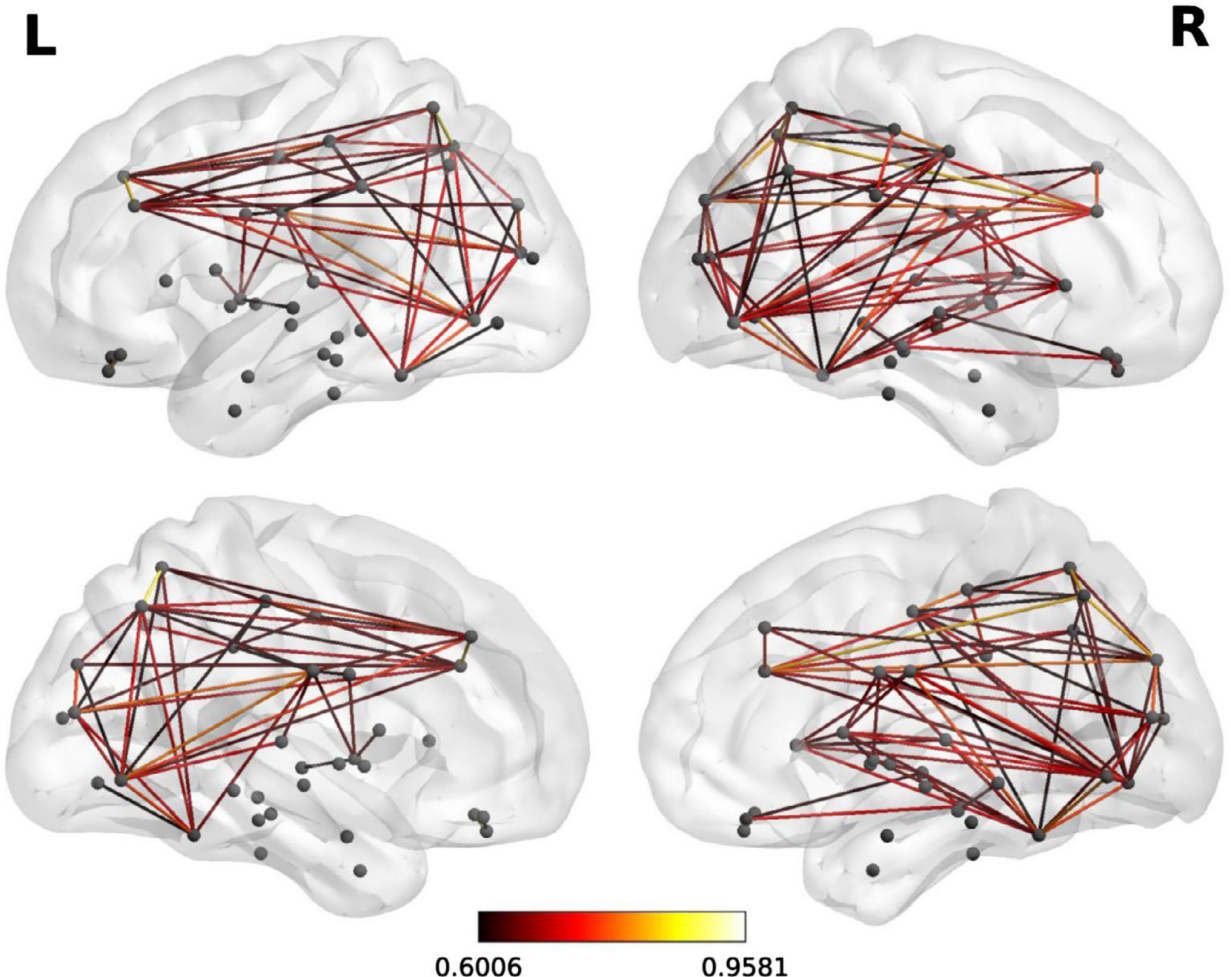
A representative functional brain network from the same participant shown in Figure 1 during the multi-sentence reading comprehension task. Notes: The analysis used a threshold at 10% sparsity. Colors correspond to *r*-values. This figure was generated using BrainNet viewer (https://www.nitrc.org/projects/bnv).

Two versions of this clustering measure exist: the global which gives an overall indication of the clustering in the network and the local which gives an indication of the embeddedness of single nodes. The main graph theory values reported are the *local clustering coefficients* which come from of a vertex (node) from our set of 68 cortical brain regions (in which case each brain region is tested as a node) and quantifies how close its neighbors are to being a clique (complete graph). Figure 1 is an example of a correlation matrix which was used in the graph theory analysis from one of the typical readers in the control group. Figure 2 is an example of functional brain network from a single participant.

Mixed ANOVAs for time, diagnostic group, and interaction between time and group were then performed on the clustering coefficient outcomes that resulted from the graph analyses. Results are reported in Table 2.

#### 2.5.3. Correlations of white matter DTI indicators with gray matter clustering coefficients with white

In related study about brain RTI for writing (Richards et al., 2017) DTI parameters were analyzed from seed points in left precuneus—in the default network and rich club network of connectionist models (Sporns, 2013; Van den Heuvel & Sporns, 2011) or left occipital temporal, left supramarginal, and left inferior frontal identified in a metaanalysis for written words (Purcell, Turkeltaub, Eden, & Rapp, 2011). FSL’s probabilistic tractography and bedpost software had been used to generate the tracts that were connected to the seed regions and FSL software randomize had been used to correct for multiple comparisons (see Richards et al., 2015).

Three of the DTI indicators of white matter integrity that had shown significant time effects at *p* < .001 from before to after instruction in Richards et al. (2017) were correlated with the gray matter clustering coefficients in the current study: axial diffusion (AD) in left superior frontal, mean diffusivity (MD) in left superior corona radiata, and MD in left middle frontal gyrus. Axial Diffusivity (AD), which is diffusivity along and parallel to the principal axis, has been associated with the axon diameter (Song et al., 2002, 2005; but see Mori, 2007, for a critical perspective on this interpretation). See Basser and Pierpaoli (1996), Mori (2007), and Wheeler-Kingshott and Cercignani (2009) for additional information on diffusivity including mean diffusivity (MD).

## 3. Results

### 3.1. Set 1 research questions: Behavioral results for RTI

As shown in Table 1, there were significant diagnostic group effects and time effects on word-level achievement measures related to the hallmark impairment in dyslexia—oral reading of real words and pseudowords and word spelling—and time effects. Eta square showed mostly modest effects size. For all measures, the two reading disabilities groups scored lower than the two control groups. Importantly, all four groups showed significant improvement from before to after the instructional intervention (see Table 1). However, despite the lack of time × diagnostic group interactions at the behavioral level of analysis, at the brain level of analysis the results for the brain’s response to the same multi-level reading instruction, which are reported next, identified significant time × diagnostic group interactions for the clustering coefficients in brain connectivity.

### 3.2. Set 2 research questions for fMRI connectivity clustering coefficients RTI

#### 3.2.1. Word level reading

Only the time × diagnostic group interaction in right medial cingulate gyrus was significant. Functional connectivity in right medial cingulate gyrus decreased in magnitude in the two control groups, but increased in magnitude in the two reading disabilities groups from before to after instructional intervention (see Table 2).

#### 3.2.2. Single-sentence level reading comprehension

In both the left superior frontal gyrus and left inferior frontal gyrus, there were significant group × time interactions. Following instructional intervention, functional connectivity decreased in magnitude in the two control groups, but increased in magnitude in the two reading disabilities groups. In the left middle gyrus, there was only a significant group effect. The magnitude of connectivity was highest in dyslexia, next highest in OWL LD, next highest in dysgraphia, and lowest in the typical reading and writing controls. In the right lateral cingulate gyrus, there was only a significant time effect. Following instructional intervention, all groups changed but variably with magnitude of connectivity increasing or decreasing in both the control groups and the reading disability groups (see Table 2).

#### 3.2.3. Multi-sentence level reading comprehension

A significant main effect for time occurred in the left insula. Following instructional intervention, all groups changed but variably with magnitude of connectivity increasing or decreasing in both the control groups and the reading disability groups. In right middle frontal gyrus, there was a significant time × diagnostic group interaction, which had the highest effect size at the brain level of analysis. Both control groups increased and both reading disabilities groups decreased in magnitude of functional connectivity. The OWL LD decreased the most (aee Table 2).

### 3.3. Set 3 research questions: Correlations between DTI indicators and fMRI clustering coefficients before and after instruction

The correlations were performed on the whole sample because power was not sufficient for correlations for single diagnostic groups. Only DTI diffusivity measures were included in these analyses that had shown significant time effects at *p* < .001.

#### 3.3.1. Word reading judgments

At time 1, AD in left superior frontal white matter was not correlated with the clustering coefficient in right inferior frontal gyrus; but at time 2, AD in left superior frontal white matter was correlated with the gray matter clustering coefficient in right inferior frontal gyrus, *r* = .32, *p* = .046, on this word-level silent reading task. Their correlation became significant only after instructional intervention.

#### 3.3.2. Single-sentence reading comprehension

At time 1, MD in left superior corona radiata was not correlated with the clustering coefficient in left middle frontal gyrus; but at time 2, MD in left superior corona radiata was correlated with the clustering coefficient in left middle frontal gyrus, *r* = .35, *p* = .03, on this syntax-level silent reading task. Again, their correlation became significant only after instructional intervention. can be used to interpret both group effects and time × diagnostic group effects.

#### 3.3.3. Multi-sentence reading comprehension

At time 1, MD in left anterior corona radiata was not correlated with the cluster coefficient in right middle frontal gyrus; but at time 2, MD in left anterior corona radiata was correlated with cluster coefficient in right middle frontal gyrus, *r* = .33, *p* = .04, on this multi-sentence level silent reading task. The correlation became significant after instruction.

## 4. Discussion

### 4.1. Set 1 research questions

There was evidence for Behavioral RTI. All the groups responded from before to after completing the computerized lessons in three word level skills related to oral and silent reading. This finding is encouraging because it suggests that those with reading disabilities can improve on skills related to their hallmark deficits. However, those with dyslexia and OWL LD did not read words at the same achievement levels as control groups either before or after the intervention. Thus, in evaluating RTI it is important to consider both whether RTI was observed and whether RTI was comparable for those with and without reading disabilities. If those with reading disabilities do not reach comparable levels as those without reading disabilities, then additional instructional intervention is warranted until they do. Thus, it is important to assess response to intervention in both those with reading disabilities and those without reading disabilities.

### 4.2. Set 2 research questions

There was evidence of Brain RTI. Clustering coefficients in brain regions analyzed in reference to the CO network involved in cognitive control varied according to the level of language involved in the fMRI reading tasks—words, sentences, or multi-sentence text—for which there were significant time × group interactions. Right medial cingulate was involved at the lexical/word level. Left inferior frontal and left superior frontal gyri were involved at the syntax/single sentence level. Right middle frontal gyrus was involved at the text/multi-sentence level. These time × diagnostic group interactions identified treatment effects specific to diagnoses and levels of language and show that the brain’s response to multi-level reading instruction is unlikely due to simple placebo effects. Not only presence versus absence of a reading disability or nature of a reading disability but also level of language in a reading task may affect conclusions about the reading brain’s RTI.

### 4.3. Set 3 research questions

The correlations between gray matter and white matter measures observed after instruction, but not before instruction, also varied for fMRI reading tasks at different levels of language. Prior findings of fMRI studies that associated BOLD activation in a ROI with specific functions in children with and without dyslexia offer potential interpretations of the current findings for fMRI connectivity. At the *lexical (word) level*, AD in left superior frontal white matter was correlated with the clustering coefficient in right inferior frontal gyrus, a region where BOLD activation was related to orthographic coding of written words (Aylward et al., 2003). At the *syntax (sentence) level*, MD in left superior corona radiata was correlated with the clustering coefficient in left middle frontal gyrus, a region where BOLD activation was related to working memory during written word tasks (Richards, Berninger, & Fayol, 2009). At the text level (multi-sentence) level, MD in left anterior corona radiata was correlated with right middle frontal gyrus, a region BOLD studies also showed is involved in working memory for written words (Richards et al., 2009). The current findings also show the involvement of the right middle frontal gyrus in processing sequential sentences containing multiple written words to comprehend text that requires inferencing (going beyond what is stated in the written sentences).

Collectively, these significant white matter–gray matter correlations that emerged after instructional intervention are consistent with prior research findings of joint maturation of white matter gray matter and gray matter connections in fronto parietal pathways (Olesen, Nagy, Westerberg, & Klingberg, 2003). That these emerging correlations were significant for measures of DTI diffusivity is also consistent with prior research showing changes in white matter following intervention; for example, in the fornix (Hofsteller, Tavor, Moryosef, & Assaf, 2013). However, the current study extends these findings about white matter-gray matter relationships to students with and without reading disabilities.

### 4.4. Limitations, future directions, and conclusions

One limitation was having only behavioral measures of word reading and not syntax reading in the age levels studied; the reading comprehension measures used in the intervention study confounded sentence and text reading comprehension. Future research might employ separate behavioral measures of sentence level and text level comprehension to compare OWL LD and dyslexia as well as the reading disabilities groups with control groups.

Another limitation was that the sample size is relatively small for each of the diagnostic groups, but not necessarily smaller than in other studies of brain bases of reading disabilities. Additional studies are therefore needed to evaluate if findings replicate across samples of students with and without reading disabilities.

It is also the case that findings may depend on fMRI tasks employed. Thus, it is an important finding of the current study that for the second and third set of research questions the results varied across three levels of language (word, syntax/sentence, and multi-sentence text). Future research might continue to address the issues of how (a) functional connectivity based on clustering coefficients for networks involved in cognitive control may vary according to level of language involved in language tasks participants are asked to perform while their brain is imaged; (b) the level(s) of language taught in instruction after a first brain scan and before a second brain scan; and (c) brain response following instruction compared to before instruction of students with and without reading disabilities defined by hallmark impairments at different levels of language.

For an organ as complex as the human brain with multiple levels ranging from molecular to emergent functional systems along bottom-up, top-down, and right-left axes, and as plastic as the brain may be across development and in response to learning environments, the results of the current study are heartening. Even in students with persisting reading disabilities, gray matter clustering coefficients related to the CO network involved in cognitive control of the reading brain changed at multiple levels of language during reading *and* new white matter-gray matter relationships emerged in response to reading instruction. Both behavioral and brain data can contribute to understanding of the complex functional reading system and inform practices to help optimize reading achievement in students with diagnosed specific reading disabilities.

## Figures and Tables

**Table 1:** Significant behavioral effects in word reading achievement from before to after computerized lessons

Test	Time 1		Time 2		(df)	*F*	*p*	*η^2^*
	*M*	(SD)	*M*	(SD)				
*TOC sight spelling*								
Time	8.75	(.48)	9.44	(.50)	(1, 28)	4.51	<.05*	.14
Group					(3, 28)	8.85	<.000***	.49
Control	12.20	(1.10)	12.40	(2.28)				
Dysgraphia	10.00	(.3.25)	11.25	(2.31)				
Dyslexia	7.46	(2.15)	7.92	(2.72)				
OWL LD	5.33	(2.50)	6.17	(2.40)				
Time × Diagnostic group					(3, 28)	.51	.66	.05
*TOWRE sight*								
Time	93.73	(16.16)	96.61	(17.68)	(1,36)	5.42	<.05*	.13
Group					(3, 36)	6.36	.001***	.35
Control	100.86	(11.95)	106.86	(12.79)				
Dysgraphia	106.78	(18.16)	107.67	(22.03)				
Dyslexia	70.41	(12.99)	93.76	(13.42)				
OWL LD	78.29	(9.09)	80.70	(11.88)				
Time × Diagnostic group					(3, 36)	.62	.61	.05
*TOWRE phonemic*								
Time	91.23	(2.43)	94.87	(2.53)	(1, 36)	10.58	<.01**	.23
Group					(3, 36)	7.64	<.001***	.39
Control	101.57	(12.33)	106.14	(14.04)				
Dysgraphia	104.00	(19.67)	106.33	(20.14)				
Dyslexia	86.06	(14.22)	87.29	(12.91)				
OWL LD	73.29	(6.45)	79.71	(11.10)				
Time × Diagnostic group					(3, 36)	1.14	.33	.09

Notes: *TOC sight spelling* (Mather et al., 2008) assesses word-specific spelling. *TOWRE* (Torgesen et al., 1999) assesses accuracy and rate of orally reading real words (*TOWRE sight*) and nonwords (*TOWRE phonemic*). Only results for each group at each time rather than for each group across the two times are reported to interpret both group and time × group effects.

* *p* < 0.05.

** *p* < 0.01.

*** *p* < 0.001

**Table 2:** Graph analysis clusters for fMRI connectivity (gray matter) on reading tasks

fMRI task and regions	Time 1 *M* (SD)	Time 2 *M* (SD	*F*(df)	*p*	*η^2^*
Word-specific reading/spelling					
*Right medial cingulate gyrus*					
Time	.076 (.013)	.061 (.011)	(1, 39) .89	.35	.02
Group			(3, 39) 1.06	.38	.08
Control	.148 (.17)	.050 (.026)			
Dysgraphia	.068 (.060)	.061 (.088)			
Dyslexia	.045 (.042)	.082 (.069)			
OWL LD	.044 (.051)	.050 (.045)			
Time × Diagnostic group			(3,39)3.42	<.05*	.21
Single Sentence Reading with and without homonym foils					
*Left superior frontal gyrus*					
Time	.219 (.032)	.162 (.028)	(1,39)1.97	.17	.05
Group			(3,39)1.08	.37	.08
Control	.383(.357)	.139 (.145)			
Dysgraphia	.207(.142)	.093 (.101)			
Dyslexia	.146 (.145)	.196 (.172)			
OWL LD	.140 (.111)	.219 (.243)			
Time × Diagnostic group			(3, 39)3.40	<.05*	.21
*left middle frontal gyrus*					
Time	.108 (.025)	.119 (.039)	(1,31).05	.82	.00
Group			(3, 39)5.27	<.01**	.29
Control	.053 (.056)	.053 (.084)			
Dysgraphia	.083 (.047)	.085 (.073)			
Dyslexia	.211 (.033)	.240 (.052)			
OWL LD	.083 (.060)	.098 (.094)			
Time × Diagnostic group			(3, 39) .03	.99	.00
*Left inferior frontal gyrus*					
Time	.302 (.060)	.143 (.044)	(1, 39) 6.50	<.05*	.14
Group			(3, 39)1.47	.24	.10
Control	.625 (.709)	.139 (.345)			
Dysgraphia	.311 (.349)	.103 (.164)			
Dyslexia	.192 (.193)	.211 (.297)			
OWL LD	.082 (.110)	1.20 (.087)			
Time × Diagnostic group			(3, 39) 3.81	<.05*	.23
*Right lateral cingulate gyrus*					
Time	.022 (.008)	.095 (.038)	(1, 39) 4.68	<.05*	.11
Group			(3, 39) .94	.43	.07
Control	.061 (.045)	.081 (.086)			
Dysgraphia	.422 (.907)	.053 (.066)			
Dyslexia	.103 (.081)	.141 (.391)			
OWL LD	.043 (.031)	.037 (.033)			
Time × Diagnostic group			(3,39) .16	.69	.00
Multi-sentence reading					
*Left insular region*					
Time	.005 (.004)	.050 (.022)	(1, 39) 4.17	<.05*	.10
Group			(3, 39)1.47	.24	.10
Control	.000 (.000)	.000 (.000)			
Dysgraphia	.038 (.111)	.008 (.023)			
Dyslexia	.048 (.140)	.009 (.031)			
OWL LD	.001 (.003)	.107 (.193)			
Time×Group			(3,39) 2.06	.12	.14
*Right middle frontal gyrus*					
Time	.223 (.035)	.215 (.054)	(1, 39) .02	.90	.00
Group			(3, 39)2.00	.13	.13
Control	.043 (.042)	.433 (.660)			
Dysgraphia	.082 (.092)	.197 (.264)			
Dyslexia	.155 (.183)	.148 (.178)			
OWL LD	.610 (.445)	.080 (.091)			
Time × Diagnostic group			(3, 39) 6.71	.001***	.34

Notes: Only results for each group at each time rather than for each group across the two times are reported. These can be used to interpret both group effects and time × diagnostic group effects.

* *p* < 0.05.

** *p* < 0.01.

*** *p* < 0.001

## PUBLIC INTEREST STATEMENT

Some individuals struggle more than others in learning to read and not all reading disabilities are the same. In this study, an interdisciplinary team investigated how students who did and who did not have persisting reading disabilities during middle childhood or early adolescence responded to instruction. Both those who did and did not improved significantly on word reading achievement measures even though those with reading disabilities did not catch up to those without reading disabilities after just 18 lessons. They also showed two kinds of significant brain changes at different levels of language: words, sentences, and text. First, both those with and without reading disabilities changed in clustering coefficients (networks) in specific regions from before to after instruction. Second, correlations between white matter and gray matter emerged only after instruction and instruction may have enabled the white matter to connect with the gray matter clustering. Teaching changes the reading brain!
